# Geodiversity in the Amazon drainage basin

**DOI:** 10.1098/rsta.2023.0065

**Published:** 2024-04-01

**Authors:** Cécile M. E. Alsbach, Arie C. Seijmonsbergen, Carina Hoorn

**Affiliations:** Institute for Biodiversity and Ecosystem Dynamics (IBED), University of Amsterdam, PO Box 94240, 1090GE, Amsterdam, The Netherlands

**Keywords:** Amazonia, geodiversity, soils, geology, geomorphology, hydrology

## Abstract

The Amazon is the largest drainage basin on Earth and contains a wide variety of abiotic landscape features. In spite of this, the geodiversity in this basin has not yet been objectively evaluated. We address this knowledge gap by combining a meta-analysis of an existing global geodiversity map and its components with a systematic literature review, to identify the key characteristics of geodiversity in the Amazon drainage basin (ADB). We also evaluate how these global geodiversity component maps, that are based on the geology, geomorphology, soils and hydrology, could be refined to better reflect geodiversity in the basin. Our review shows that geology—through lithological diversity and geological structures—and hydrology—through hydrological processes that influence geomorphology and soil diversity—are the main determinants of geodiversity. Based on these features, the ADB can be subdivided into three principal regions: (i) the Andean orogenic belt and western Amazon, (ii) the cratons and eastern Amazon, and (iii) the Solimões-Amazon river system. Additional methods to map geomorphological and hydrological diversity have been identified. Future research should focus on investigating the relationship between the geodiversity components and assess their relationship with biodiversity. Such knowledge can enhance conservation plans for the ADB.

This article is part of the Theo Murphy meeting issue ‘Geodiversity for science and society’.

## 1. Introduction

Geodiversity has been described as the ‘abiotic equivalent of biodiversity’ [1] or more precisely ‘…the natural range (diversity) of geological (rocks, minerals, fossils), geomorphological (landform, processes) and soil features, … [including] their assemblages, relationships, properties, interpretations and systems’. [2]. The processes that created geodiversity are key to the natural dynamics in a landscape and their protection warrants the functionality of terrestrial and marine systems, and the conservation of habitats and landscapes [3]. Conserving geodiversity is thus of great importance as, despite minor exceptions, restoration of geodiversity is nearly impossible [1].

In recent years, the concept of geodiversity has received special attention in relation to patterns of biodiversity and biogeography [4–9]. The perceived link between abiotic and biotic diversity (geodiversity and biodiversity, respectively) is based on the notion that areas with high geodiversity drive speciation and support high biodiversity [10,11]. This view has now been corroborated by research [12–14], though there are examples of areas with high geodiversity containing low biodiversity, such as mountain ranges (which are high in topographic diversity) located in hot desert climates or Arctic regions [4]. Moreover, the role of climate in driving speciation should not be ignored either [15]. Despite these objections to a strict relationship between geodiversity and biodiversity, preserving geodiversity offers a potentially important approach to conserving biodiversity in the face of environmental and climate change, a concept that has become known as ‘conserving nature's stage’ [11,16,17].

Conserving geodiversity is also essential when developing nature-based solutions for global challenges regarding the demand for natural resources, especially those related to human well-being and ecosystem functioning [5]. Thus, failing to conserve geodiversity jeopardizes not only the existence of the geofeatures themselves, the landscape they are a part of and the geosystem services they provide, but also the continued existence of the abiotic conditions (i.e. habitats) that biotic elements (i.e. organisms) depend on [3,10,18]. Finally, conserving geodiversity is also important for safeguarding geological archives, to ensure the possibility of future scientific research and to capitalize on the potential touristic value of a landscape and conserve cultural values assigned to geofeatures [1,3,19,20].

**Figure 1.**
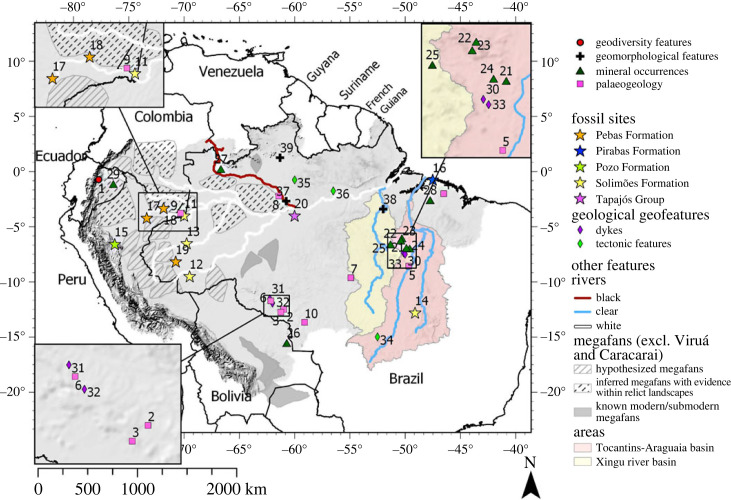
Map showing the extent of the ADB, *sensu* *lato*, according to Albert *et al*. [21], as well as areas and locations mentioned in the text and country names. See table 2 for the locations’ names and corresponding articles. Tocantins-Araguaia and Xingu river basins were retrieved from Venticinque *et al*. [22]. Locations of the megafans, excluding Viruá and Caracarai megafans, were retrieved from Wilkinson *et al*. [23] and Latrubesse *et al*. [24]. (Online version in colour.)

Here we will focus on the Amazon drainage basin (ADB) (figure 1), an area known for its exceptional biodiversity and ranking among the most biodiverse on Earth [25–27]. Geodiversity may have contributed to the development of such high rates of biodiversity. Therefore, understanding the development of geodiversity is valuable for the conservation of biodiversity, both on a local level as well as on a global level [16]. Until now, research on geodiversity components (i.e. geology, geomorphology, topography, hydrology and soils [9]) in the Amazon landscape has mostly been confined to biogeographic research (e.g. [28–30]) and research assessing the relationship between topographic complexity, climatic variability and biodiversity in the northern Andes [31]. Other studies have addressed individual geodiversity components and the interplay between these components and geodiversity in the ADB [32–34], but an objective assessment of geodiversity in the basin at large is still lacking.

Most of geodiversity-related research in the ADB focused on smaller extents within the ADB, such as individual countries [35,36], a predefined area of the ADB [37] or smaller watersheds within the ADB [38]. In addition, comparison of geodiversity across the literature is difficult, because different methods have been used to either describe or quantify geodiversity [3,10,39].

In this study, we identify the key characteristics of geodiversity and its components. To do this, we use the recently published global quantification of geodiversity metrics by Polman *et al*. [40], a meta-analysis of local and regional maps of geodiversity components in the ADB (geology, soils, geomorphology and hydrology), combined with a systematic literature review of geodiversity in the ADB. We also investigate if the global geodiversity map should be amended to more accurately reflect geodiversity in the ADB. Together, these data enabled us to generate a comprehensive understanding of geodiversity development across the ADB.

## 2. Methodology

The study area is the ADB (*sensu* *lato*) as defined by Albert *et al*. [21] (figure 1). The digital areal extent of the ADB was provided by a hydrological study in the ADB [22], and was used to extract all relevant data. To assess the geodiversity in the ADB, we combined a meta-analysis of global, regional and local geodiversity index and component maps, and a systematic literature review on geodiversity. The geodiversity maps for the ADB were clipped from available global maps [41] of geodiversity components [40] that were created using openly available sources [42–45]. The spatial arrangements of the four geodiversity components (lithology, soil, geomorphology and hydrology) were ranked using zonal statistics to create diversity maps for each component. In this paper, we present the geodiversity maps and their base maps for the ADB. The basemap for hydrology is an exception, because of the density of river channels in the source data [45] prevents proper visualization on a small map. Therefore, we include a map of the three major river types (blackwater, clear water and whitewater rivers) following Venticinque *et al*. [22]. All maps are overlain with the South American country boundaries [46]. For all map processing routines, ArcGIS Pro 2.2.0 was used [47]. Table 1 presents an overview of the data that were used for the construction of the maps. All data that were used in the analysis and interpretation of the geodiversity and the geodiversity component maps, as well as the data used for other maps in this review are made available through an ArcGIS project package file (see electronic supplementary material) [51].

**Table 1.** Table listing sources and processing steps for each map. All processing steps were performed in ArcGIS Pro 2.2.0 [47].

data	reference	processing
extent ADB, river type map	Venticinque *et al*. [22]	retrieved feature polygons within the extent of the ADB polygon feature
geodiversity index, lithological, slope, hydrological and soil diversity index maps	Polman *et al*. [41]	maps provided in the database were clipped using the ADB extent
lithological map	Hartmann & Moosdorf [42]	data were retrieved using Esri Living Atlas service and clipped to ADB extent
digital elevation map (DEM) and hillshade map	Yamazaki *et al*. [43]	DEM raster data were projected to the South America Albers Equal Area Conical Projection, and clipped to ADB extent. The DEM was then used to create a hillshade map
soil type map	Hengl *et al*. [44]	data retrieved using a WCS server connection from https://maps.isric.org, and clipped to the ADB extent. Soil types with counts lower than 750 000 were grouped in a new category, ‘Other’
country boundaries	US Department of State Office of the Geographer [46]	retrieved polygons, data required no further processing
sedimentary basins map	Albert *et al*. [48], Klein *et al*. [49]	original maps were georeferenced and traced
extent megafans	Wilkinson *et al*. [50], Latrubesse *et al*. [24]	original maps were georeferenced and traced

**Table 2.** Locations mentioned in the text with their corresponding ID in figure 1.

type		ID	location
geodiversity features		1	Napo Sumaco Aspiring Geopark [53]
palaeogeology		2	Anarí Formation [54]
		3	Trincheira Complex [55]
		4	Gurupi Belt [49]
		5	Araguia Belt [56]
		6	Nova Brasiândia-Aguapeí Belt [57]
		7	Colider Suite [58]
		8	Mucajai AMG Complex [59]
		9	Los Chorros site [60]
		10	quarries in support of Snowball Earth hypothesis [61]
fossil sites	Solimões Fm.	11	fossil site Solimões Formation: freshwater bivalves, gastropods, crocodilian coprolites [62]
		12	fossil site Solimões Formation: *Purussaurus brasiliensis* [63]
		13	fossil site Solimões Formation: ostracods, fish remains, gastropods, bivalves, gyrogonites [64]
		14	fossil site Solimões Formation: crocodilians, rodents, xenarthrans, charachid fishes, turtles, birds, squamates, litopterns, primates [65]
	Pozo Fm.	15	fossil site Pozo Formation: *Neognathodus* [66]
	Pirabas Fm.	16	fossil site Pirabas Formation: benthic foraminifera, bryozoans, calcareous algae, echinoderms, molluscs, crustaceans, ichnofossils and fish assemblages [67]
	Pebas Fm.	17	fossil site Pebas Formation: glochidia [68]
		18	fossil site Pebas Formation: molluscs [69]
		19	fossil site Pebas Formation: ichnofossils [70]
	Tapajós Group	20	fossil sites Tapajós Group: *Neognathodus* [71]
mineral occurrences		21	Lago Grande Complex [72]
		22	Grão Pará Group [73]
		23	Águas Claras Formation [74]
		24	Canaã and Rio Maria region [75]
		25	Jaguar hydrothermal nickel sulfite deposit [76]
		26	Cerro Manomó [77]
		27	Morro dos Seis Lagos [78]
		28	Paragominas-Capim kaolin bauxite district [79]
		29	salt lick [80]
surface expressions of geological processes	dykes	30	mafic dykes, Carajás region [81]
		31	mafic dykes, central-east Rondônia [82]
		32	mafic dykes, Nova Brasilândia belt [83]
		33	mafic dykes, Carajás region [84]
	tectonic features	34	thrust and fold belt, Planalto da Serra [85]
		35	fractures and shear indicators, Pitinga mining district [86]
		36	neotectonic activity, Porto Trombetas [87]
geomorphological geofeatures		37	Anavilhanas Archipelago [88]
		38	Volta do Xingu [89]
		39	Viruá and Caracarai megafan [90–93]

Each map was visually evaluated for patterns in the diversity metrics, which were then interpreted in the environmental context of the ADB. A systematic literature review was conducted to supplement the interpretation of the maps, and to assess whether patterns identified in the global geodiversity maps agreed with existing literature on the ADB. The following search terms were entered into Scopus on 6 May 2023 after creating an inventory of terms closely associated with geodiversity and the ADB:

(TITLE-ABS-KEY (amazon * AND (geodiv * OR geological AND (site OR heritage OR park OR environment OR framework OR element) OR geosite OR geoheritage OR geopark OR geoenvironment OR geoconservation OR ((geodiv * OR geoheritage) AND element))) AND NOT TITLE-ABS-KEY (mars OR martian)) AND PUBYEAR >1989

The search was then refined to only include articles published after 1989, as it was around 1990 that the term geodiversity is used in the same way as it is defined in this review [3]. Because ‘Amazonian’ also refers to a geological period on the planet Mars [23], the condition to not include ‘mars OR martian’ was added to the final search. This search yielded 399 articles, which were assessed in two rounds. In the first round, articles were eliminated based on titles and abstracts. Articles were only included if: (i) the ADB is discussed and (ii) the geodiversity, one or more geodiversity components, geofeatures or potential geosites (rock formations, fossil sites, mines, landscape units, rare river features, soil types, abiotic gradients across the landscape (i.e. differences in the concentration of certain compounds or pH across water bodies or soils), or geological, geomorphological, hydrological or soil processes) are discussed. In the second round, the full text of the remaining articles was screened for information that is contributory to our aims. The inclusion and exclusion of studies were tracked using a PRISMA flow diagram [52]. An overview of geofeatures or areas otherwise interesting with regards to geodiversity was produced (table 2) and mapped (figure 1). In electronic supplementary material, S1, a full overview of the articles that were assessed, eliminated and the reasons for elimination are listed. Additional searches were conducted if the resulting information was deemed inadequate to fully explain the patterns depicted in the diversity metric maps.

## 3. Geodiversity in the Amazon drainage basin

Prior to describing the geodiversity components of the ADB, we will first assess the general geodiversity patterns in the ADB.

**Figure 2.**
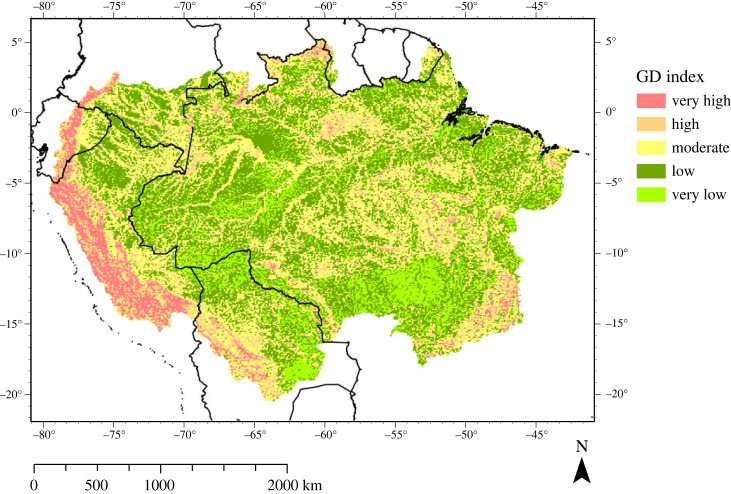
Distribution of geodiversity in the ADB. This map is based on calculating and adding separate indices of geology, topography, soil types and hydrology (table 1), and presenting geodiversity index scores in five classes (very low to very high) using a Jenks natural breaks classification [41]. (Online version in colour.)

**Figure 3.**
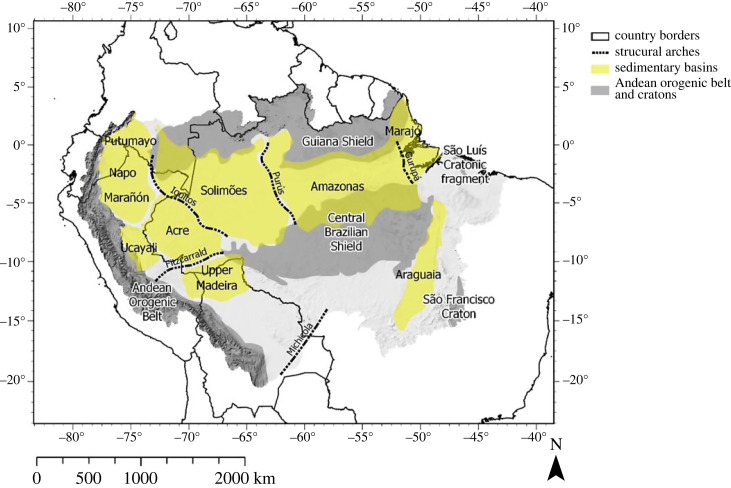
Map showing the major sedimentary basins, AOB, cratons and structural arches in the ADB. Map created after Albert *et al*. [48], used with permission from the first author. Location of the São Luís cratonic fragment retrieved from Klein *et al*. [49]. (Online version in colour.)

There are several areas with distinctly higher geodiversity index classes across the ADB (figure 2), the most prominent of these being the Andean orogenic belt (AOB), that stretches in north/south direction across Colombia, Venezuela, Ecuador, Peru and Bolivia. Two other areas of distinct geodiversity are the Guiana Shield and Central Brazilian Shield, which are respectively the northern and southern extrusions of the Amazon Craton [94]. These shields are separated by the Solimões-Amazon river system, that is entrenched in an ancient rift zone [94] that extends from west to east, subdividing the Amazon into a northern and southern section (figure 3). To a large extent the low geodiversity in the sub-Andean zone, and from west to east across the drainage basin, is explained by subsurface structures, such as sedimentary basins and structural arches [34,48,94,95] (figure 3).

The geodiversity index classes across the ADB are generally lower than in the AOB and in the Amazon Craton, but there is a pattern of moderate to high geodiversity classes reminiscent of a network of river channels extending from the craton towards the AOB. When following the rift zone, that subdivides the Amazon Craton, we see a band of moderate to high geodiversity that coincides with the location of the Solimões-Amazon river system (figures 1 and 2). This band expands into a network of channels at the border region between Brazil, Colombia and Peru, intersecting an area of very low geodiversity towards the AOB. In eastern Ecuador and southeastern Peru, at the foot of the AOB, the fluvial network reaches fan-like shapes, which correspond to the alluvial megafans that are situated in the sub-Andean foreland basins on the eastern margin of the AOB (figures 1 and 3) [50,94]. The high geodiversity pattern in the southeastern part of the ADB corresponds to the northeastern part of the São Francisco Craton [96]. The coastal areas generally contain very low to low values of geodiversity, with the exception of the Guiana coastal region and south of the city of São Luís, where the São Luis Craton is located [49] (figure 3). Lastly, there is a distinct area of very low geodiversity south of the Central Brazilian Shield, which coincides with the head of the Xingu river catchment and the Tocantins-Araguaia river system.

### (a) Previous geodiversity studies in the Amazon drainage basin

Only few papers have described the geodiversity in the ADB until now, including four papers that discuss geodiversity in the Amazon, namely on the ‘Amazônia legal’ in Brazil [37] (the northern states of Brazil, including Mato Grosso and the west of Maranhão), the Xingu river basin [38], French Guiana [35] and the NAPO Sumaco (aspiring UNESCO) Geopark [53] (figure 1). De Andrade *et al*. [37] conducted an extensive literature study of geodiversity in the ‘Amazônia legal’ and concluded that there is still little known about geodiversity in this region, neither is there information on a detailed scale. The majority of the studies emphasize the geotourism potential, the importance of geoconservation and suggest the establishment of geoparks, although the terms geodiversity and geotourism were not always conceptualized or used within a methodological framework. The summary of geodiversity indices that were used in this work includes interesting geofeatures, such as the geotourism potential of waterfalls, caves and archaeological sites. When seen in the context of the geodiversity components considered by Polman *et al*. [40], geology and fluvial geomorphology stand out as important themes within the characterization of geodiversity of the ‘Amazônia legal’.

The geodiversity studies of the Xingu river basin and French Guiana were conducted using a grid-based approach similar to the study by Polman *et al*. [40], but considered different components to define geodiversity. Instead, Scammacca *et al*. [35] considered mineral diversity alongside lithological, hydrological and geomorphological diversity. They found that geodiversity was mainly controlled by lithological diversity, and that clusters of high geodiversity classes occurred along mineral rich deposits. Silva *et al*. [38] also used palaeontological occurrences and mineral diversity as alternative components of geodiversity in their Xingu river basin assessment, alongside geological, geomorphological and pedological indices. Lithological diversity was considered to be an important driver of geodiversity, by providing a variety of parent materials, resulting in high pedological and geomorphological diversity, respectively. Interestingly, the Xingu headwaters plateau region was classified as an area with high geodiversity [38], while this area was marked as an area of very low geodiversity on the global scale geodiversity index map (for comparison, see the southern part of the Xingu river basin in figure 1 with the corresponding location in figure 2). This indicates that the geodiversity maps can vary greatly, depending on scale, available data, and the selected diversity indices. Although both maps were created using different geodiversity components, and assessed geomorphological diversity differently from Polman *et al*. [40], both articles appointed lithological diversity as the main driver of geodiversity.

The geodiversity assessment of the Napo Sumaco park (figure 1, table 2) was performed according to the method proposed by Brilha [19] and was based on a literature review of the area, as well as interviews with experts of that area to define any geofeatures of interest [53]. This method does not quantify geodiversity using a grid-based approach but identifies geosites that can be regarded as *in situ* occurrences of geofeatures with high scientific value [19]. The majority of the geosites were deemed valuable as a result of geochemical properties of the site, or the occurrence of geological geofeatures [53], and as such highlight the importance of geology in the Napo Sumaco park.

**Figure 4.**
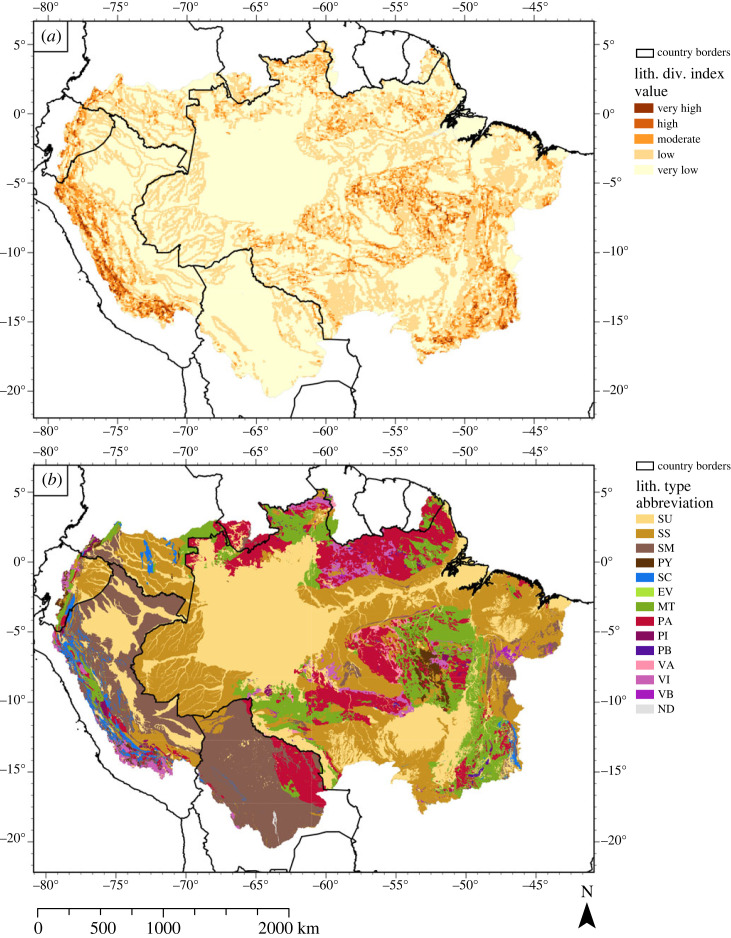
(*a*) Distribution of geological diversity in the ADB. Each 10 km × 10 km raster cell was awarded a score, based on the lithological variety in that cell. Index classes (very low to very high) were created using a Jenks natural breaks classification (see Polman *et al.* [40] for detailed information). (*b*) Map showing the most likely lithology according to Hartmann & Moosdorf [42]. SU, unconsolidated sediments; SS, siliclastic sedimentary rock; SM, mixed sedimentary rocks; PY, pyroclastics; SC, carbon sedimentary rock; EV, evaporite; MT, metamorphic rock; PA, acid plutonic rock; PI, intermediate plutonic rock; PB, basic plutonic rock; VA, acid volcanic rock; VI, intermediate volcanic rock; VB, basic volcanic rock; ND, no data. (Online version in colour.)

## 4. Geodiversity components in the Amazon drainage basin

### (a) Geological diversity

In our meta-analysis, geological diversity is expressed as lithological diversity, i.e. the number of different lithological units present in an area. The distribution of lithological diversity is shown in two maps, a lithological index map (figure 4*a*) and a predominant lithology map (figure 4*b*). Geological diversity, however, also includes geological structures that resulted from tectonism that reshaped the landscape [3]. Our literature review of the ADB suggests that geosites characterized by ‘lithological diversity and main geological structures', ‘fossils’, ‘mineral occurrences and ores', and ‘other geofeatures and geological structures’, are important.

Below we discuss the lithological diversity maps (figure 4*a*,*b*) and the geosites identified in the literature (figure 1) following a threefold subdivision of the ADB, namely the AOB and the western Amazon, the cratons and eastern Amazon, and the Solimões-Amazon (SA) river system. After this we will discuss the other types of geological diversity.

#### (i) Lithological diversity

*(1) Eastern Amazonia*: The eastern Amazon is dominated by the Amazon, São Luís and São Francisco cratons which have a very diverse lithology. This is well reflected in figure 4*a*, which shows a variety of brown shades. The cratonic rocks were formed during the early history of the Earth [94,97,98] and range in age from *ca* 3000 to 1000 Ma [34,99–101]. The cratonic regions represent the formation, amalgamation and break-up of former supercontinents [102], with numerous examples of belts, complexes and suites that are a testament to the cratons' long and geologically dynamic history. Good examples of this early history are the Trincheira Complex, which is associated with the supercontinent Columbia [55], the Nova Brasilândia-Aguapeí Belt, which is related to the amalgamation of Rodinia [57], the Gurupi [49] and Araguia Belt [56], which are both related to the amalgamation of Gondwana, and the Anarí Formation, a remnant of the magmatic activity preceding the break-up of Pangea [54]. Other areas, such as the Colider Suite [58] and Mucajai Anorthosite-Monzonite-Granite Complex [59], are suitable for palaeomagnetic studies, allowing the reconstruction of the orientation of palaeocontinents. Sedimentary records can also provide information about palaeogeological events, such as sediments found in quarries and outcrops in Rondônia and Mato Grosso, Brazil presenting evidence for the Snowball Earth hypothesis [61]. The legacy of all these geological processes is shown in the high variety of sedimentary, pyroclastic, metamorphic and volcanic rocks. The location of all examples is indicated in figure 1 (table 2 for each location's corresponding number). For more examples of how current geological formations in the Amazon Craton relate to palaeogeology, see Cordani & Teixeira [103], who describe a host of Proterozoic accretionary belts consisting of ancient rift and suture zones, and Geraldes *et al*. [102], who describe major accretionary and collisional processes that may be correlated to supercontinent assemblies.

*(2) AOB and western Amazonia:* The lithological maps of the western and central Amazon (figure 4*a*) show predominantly dark brown colours, reflecting the lithological diversity along the lower slopes of the AOB including the foreland, and the intracratonic basins. The AOB is characterized by relatively young sedimentary, metamorphic, volcanic and plutonic rocks with ages up to 1600 Ma [34,99,100] (figure 4*b*). Albeit younger than the craton, the formation of the AOB has been a geologically and tectonically complex process throughout the Cenozoic [104,105]. These processes have affected biological evolution in the Andes [106–108], but also landscapes and environments in the Amazon [33,109,110]. The early stages of Andean uplift, during the Paleogene, are recorded in the sedimentary sequence of the AOB foreland, and in the intracratonic Solimões Basin (figure 2) (i.e. Horton [105], Roddaz [95], Vallejo *et al*. [111]). The later stages of increased Andean uplift are preserved in the Neogene sedimentary record of the aforementioned basins. Furthermore, both Neogene tectonism in the AOB and plate–mantle interactions caused increased subsidence and accommodation space in the western Amazonian sedimentary basins [109,110,112–114]. These processes determined palaeoenvironmental changes and controlled the sediment input into the ADB.

Furthermore, the orographic barrier effect led to increased precipitation along the eastern flank of the AOB, and together with marine incursions, as a result of sea level changes, further altered the palaeoenvironments [33,69,115]. The sedimentary record also suggests that orbital forcing played a role in controlling sediment deposition, resulting in cyclical sedimentation patterns [60] (see figure 1 and table 2 for the location where these sediments can be observed). During the late Miocene to Quaternary, Andean uplift intensified. In combination with global climate cooling, this led to marked lithological changes in the sub-Andean zone, ranging from sand to conglomerate-dominated deposits [116,117]. The latter deposits were formed by alluvial megafans along the lower slopes of the Andes, and into the foreland basin [24,34,50,118,119].

*(3) SA river system:* This river system originates in the AOB and displays a characteristic fan-shape pattern in the western Amazon. The floodplains of the SA river system are characterized by low lithological diversity that is formed by unconsolidated channel and floodplain deposits (yellow to light brown colours; figure 4*a*,*b*). In the central Amazon geological diversity is very low due to low topographical variety. In its eastern half, the SA river system is entrenched in the Amazon Craton, terminating in the Atlantic, where it forms a massive sediment apron known as the Amazon submarine fan [100,120]. The SA river system obtained its present configuration due to two contrasting geological processes. Firstly, in the eastern Amazon the Amazon River channel occupies an abandoned rift (i.e. aulacogen) that was formed in the Early Palaeozoic and subdivided the Amazon Craton into two [34,94,100]. During the Cretaceous, this structure was reactivated following the opening of the Atlantic (fig. 4.7, [121,122]). Together, these processes shaped the causeway for the Cretaceous proto-Amazon that originated in the craton [100]. Secondly, after intensified uplift in the Andes, increased sediment supply, and changes in basin subsidence in the western Amazon during the Neogene, the proto-Amazon coalesced with the newly formed western fluvial system (Acre phase) [100,123]. The sedimentary record of the Amazon submarine fan, in the equatorial Atlantic, forms proof of this [100,120,124].

**Figure 5.**
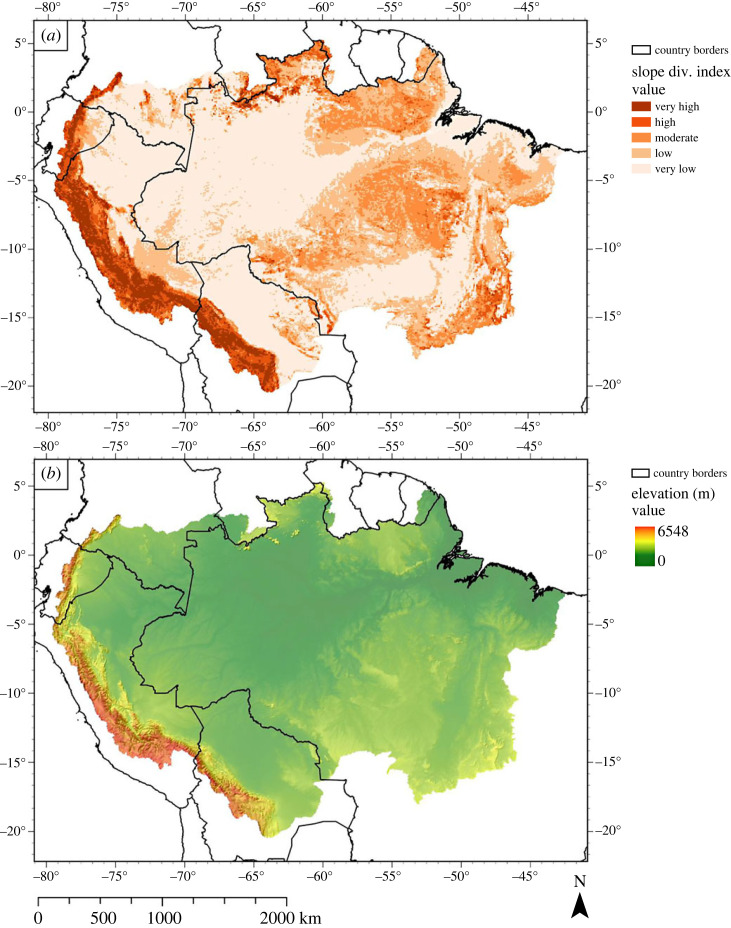
(*a*) Distribution of geomorphological diversity in the ADB. Each 10 km × 10 km raster cell was awarded a score, based on the slope diversity (calculated from the standard deviation and range of the slope) in that cell. Index classes (very low to very high) were created using a Jenks natural breaks classification (see Polman *et al*. [40] for detailed information). (*b*) Digital elevation model (DEM) of the ADB, overlaid with a hillshade. Based on data from Yamazaki *et al*. [43]. (Online version in colour.)

Despite the fact that both the slope index (figure 5*a*) and lithological diversity (figure 4*a*) in the SA river system are classified as low, Valeriano & Rossetti [125] have shown that geomorphometric data have the potential to improve geological maps by evaluating the depth of incisions in sedimentary deposits, which reflects their geological age. This also illustrates the issue of scale; while figure 4*b* only states that there are unconsolidated sediments found in the SA river system, distinctly different types of sedimentary deposits can be recognized at a finer spatial scale.

#### (ii) Other types of geological diversity in the Amazon drainage basin

As previously explained in §3a, geodiversity maps can be created by using different geodiversity components. However, there are other ways to express geological diversity than through lithological diversity. A review of the literature revealed several possible geofeatures that contribute to our understanding of geological diversity in the ADB, namely fossils, mineral occurrences and surface expressions of geological processes. All sites that are mentioned below are listed in table 2, and their locations indicated in figure 1.

The ADB contains several famous fossil sites. These sites contain fossils that are closely related to specific marine and fresh water palaeoenvironments. Most fossils originate from the Neogene, and more specifically the Miocene. Neogene deposits host a rich fossil fauna that ranges from mammals and birds [126] to crododilians and turtle fauna [127], but also a wide variety of fish fauna [128], and aquatic invertebrates [129]. Examples of Neogene fossil sites include the Pirabas [67], Solimões and Pebas formations [62–65,68,69,130], which contain many easily observable macro- and microfossils, but also trace fossils (ichnofacies) [70] and evidence of different types of marine and fluvial conditions in past eras. These sites provide evidence for quick assessment of the age and circumstances of the formation of sediment deposits [69,71], as well as understanding palaeoclimatic changes and transitions between geological eras [66], which is especially important regarding the archival function of geodiversity. The types of fossils that are found at these sites are listed in table 2.

A different type of geological diversity in the ADB is the occurrence of minerals and ores. Mineral diversity has previously been used as a factor for quantifying geodiversity in parts of the ADB [35,38] and also the many mineral deposits in the ADB deserve to be highlighted. Some examples of minerals and ores found throughout the ADB include rare earth elements, iron, nickel, sulfide, chromium and gold, which can be found in areas such as the Carajás mineral province [72–76,131], Morro dos Seis Lagos [78] and the Paragominas-Capim kaolin bauxite district [79] in Brazil, and the Cerro Manomó in Bolivia [77]. Mineral deposits may leak toxic substances, either through natural processes or through mining activity near their source [132], thereby affecting both water and soil quality. A completely different type of mineral occurrence affecting water and soil quality are natural mineral licks, which can for instance be found in the Colombian foothills [80]. Though these occurrences are quite small compared with mineral deposits, they are considered important landscape elements and as such relate to geodiversity.

Lastly, there are geofeatures that resulted from past geological processes with less palaeogeographic impact than discussed in §4a(i) or geofeatures that relate to ongoing geological processes that can be observed on a relatively fine spatial scale. We have termed these geofeatures ‘surface expressions of geological processes'. Examples of such geofeatures are the mafic dyke swarms in the Carajás Mineral Province and the state of Rondônia [81–84]. Mafic dykes represent past magmatic intrusions that, depending on weathering intensity and rock hardness, may now form positive relief, as the host sedimentary rock has weathered away. These dykes created unique geochemical conditions and may locally contribute to high geodiversity. Another example of surface expressions of geological processes are well-exposed thrust and fold belts in the Planalto da Serra [85] or fractures and shear indicators in the Pitinga mining district [86]. These geofeatures are relatively small-scale examples of how tectonic activity affects the landscape. Another example of how tectonic activity impacts the landscape is neotectonic activity in Porto Trombetas [87] and around the Negro River, affecting the rivers' (tributaries’) elevation profile [90]. These geological processes also affect the geomorphological and hydrological diversity, which stresses the interactions between different geodiversity components.

### (b) Geomorphological diversity

Geomorphological features can often be recognized by distinct elevation patterns in the landscape. Figure 5*a*,*b*, respectively, shows the slope diversity index and the elevation map (overlain with a hillshade) of the ADB. Again, a clear distinction can be made between the slope diversity index classes of the AOB (high to very high diversity), the Amazon Craton, and the São Francisco Craton (moderate to high diversity). These areas correspond to areas with a predominantly high geodiversity class in figure 2. The slope diversity index decreases sharply as one moves eastwards from the AOB. Two areas stand out as slightly higher footslope areas: east of the AOB in eastern Ecuador, which coincides with the location of the Rio Pastaza megafan [133], and southeast Peru, which coincides with the location of several hypothesized alluvial megafans [50], as well as the Fitzcarrald arch (figures 1 and 3). Alternatively, the elevated footslope areas could represent areas of uplift due to compression related to the subduction of the Carnegie and Nazca ridge beneath the South American plate [134,135].

The sub-Andean foreland and the SA river system (figure 3) have low slope diversity, and relatively low elevations (figure 5*a*,*b*). These areas also correspond to unconsolidated sediments, and likely originated from the AOB and to a lesser degree the Amazon Craton. Similarly, the Tocantins-Araguaia river system (figure 1) coincides with the depression between the Central Brazilian Shield and the São Francisco Craton. However, slope alone does not define a geomorphological unit, and as such, not all areas with a low slope diversity index class belong to the same geomorphological unit. An example is the upper reach of the Xingu river basin, a plateau-like area located west of the Tocantins-Araguaia river system, which, despite similar lithological and slope diversity (figures 4*b* and 5*b*, respectively) is highly contrasting to lowland river systems. Thus, in some instances, the slope diversity index alone does not discriminate between different geomorphological units.

Classification and mapping of landform elements based on a DEM is common practice in geomorphological mapping. Robaina *et al*. [136] have successfully used pattern recognition to distinguish different geomorphological units in the Tocantins State, Brazil, by extracting geomorphons from a DEM to reflect terrain morphology, as was proposed by Jasiewicz & Stepinksi [137]. Though their study was not conducted within the context of geodiversity, different landform units could be distinguished in the Tocantins State, even in areas that are identified as having low slope index diversity in figure 5*a*, and could therefore provide an alternative perspective on geodiversity across the ADB when applied to a larger extent.

Most geomorphological research in the ADB is conducted within the hydrogeomorphological sphere [88,90–93], with the majority of the studies located in the central Amazon. The central Amazon in general has a low geological diversity, which is likely the result of low topography, relatively low sediment input (when compared to the westernmost Amazon) and paired accommodation space [112]. Consequently, the area is dominated by sedimentary deposits (figure 4*b*) which have a low to very low slope diversity index class. Yet, these hydrogeomorphological studies distinguish several geomorphological units, such as the Viruá and Caracarai megafans (number 39 in figure 1 and table 2), alluvial plains and sedimentary basins, that provide important information regarding the areas' geological history [88,90–93]. They also identified tectonic activity as one of the main drivers for the position of current river channels, which in turn shapes fluvial geomorphology [88,90–93]. A unique example of the interaction between geological and hydrological processes is the Anavilhanas Archipelago (number 37 in figure 1 and table 2), the largest freshwater archipelago in the world [88]. Similarly, the Marajó area is also affected by subsidence and faulting which shaped the geomorphology, creating different depositional environments and drainage patterns that in turn affected vegetation patterns [138,139].

Other studies that describe morphological processes and geofeatures within river channels [140–142], mostly involve one specific river. For example, the Volta Grande do Xingu's unique and complex planform is very important for the local aquatic ecology and biodiversity [89] (number 38 in figure 1 and table 2). The building of the Belo Monte dam was expected to change riverbed characteristics [89], and drastic environmental impacts have already been reported in the news [143,144].

### (c) Hydrological diversity

**Figure 6.**
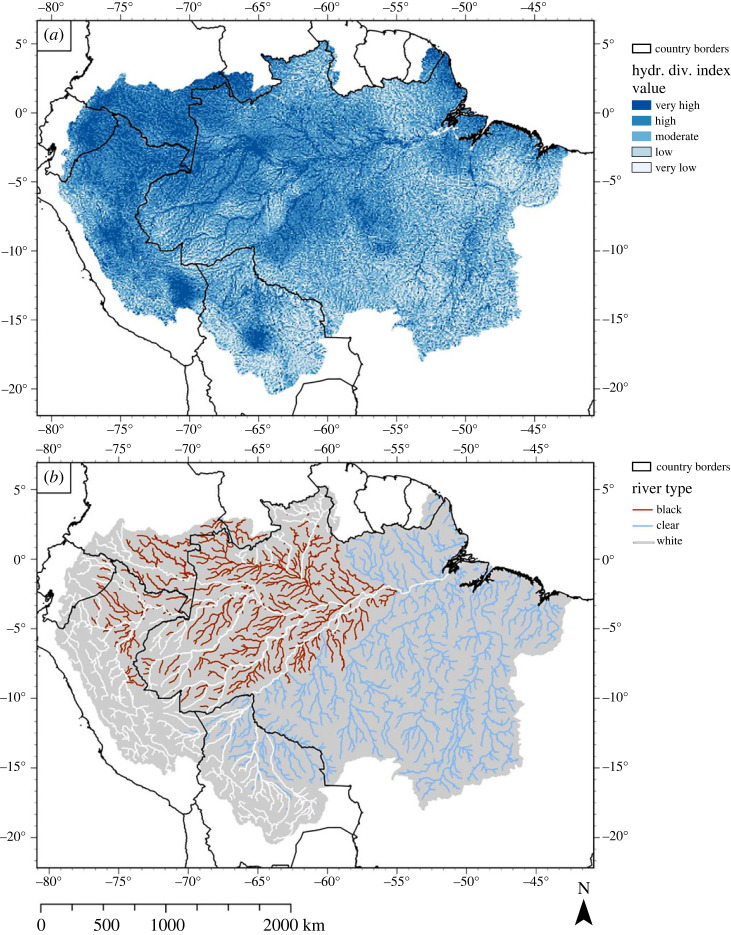
(*a*) Distribution of hydrological diversity in the ADB. Each 10 km × 10 km raster cell was awarded a score based on the hydrological variety (based on river length and lake area metrics) in that cell. Index classes (very low to very high) were created using a Jenks natural breaks classification (see Polman *et al*. [40] for detailed information). (*b*) Map showing an alternative approach to hydrological diversity, i.e. hydrogeochemical diversity, using a classification system commonly used in the Amazon drainage basin devised by Sioli [145]. Data retrieved from Venticinque *et al*. [22]. (Online version in colour.)

The global hydrological diversity map is based on metrics of the river length and lake area in a 10 km × 10 km grid [41], and it can therefore be expected to show high values near the large rivers in the ADB (figure 6*a*). In general, areas with denser drainage networks occur in areas with higher hydrological diversity, which show as moderate to high hydrological diversity classes. For example, hydrological diversity is generally higher closer to the main channels of the larger rivers across the ADB (including, but not limited to, the Negro River, the SA river system, Purus River, Juruá River, Japurá-Caquetá River and Içá-Putumayo River), on the northwestern side of the Central Brazilian Shield, and the Tocantins-Araguaia river system. Some of the river valleys are visible as relatively low-lying, elongated linear patterns in the DEM (figure 5*b*). The distribution of hydrological diversity shows some resemblance to the general trends observed in the distribution of landform dissection caused by fluvial incisions observed in the ADB; narrow and shallow channels upstream and deeper and denser channels can be found downstream [146].

The coastal areas contain moderate to very high hydrological diversity. At first thought, this could be explained by dense fluvial network in the large Amazon river delta, but this does not explain high hydrological diversity north of the Amazon delta and the area between Bélem and São Luís. Rivers originating from elevated cratons likely concentrate in low elevations, thus creating river plains with relatively high hydrological diversity (figure 6*a*).

Coupling back to the geodiversity index map in figure 2, we find that the channels of high hydrological diversity index classes occur in areas of moderate to high geodiversity in an area otherwise characterized by very low to low geodiversity index classes. For the eastern Amazon and the SA river system, as defined in §4a(i), the hydrological diversity map (figure 6*a*) even seems to be the inverse of the slope and lithological diversity. Areas that contain very low slope or lithological diversity classes generally contain high to very high hydrological diversity classes. Hydrological diversity thus seems a relatively important component of geodiversity in the ADB.

#### (i) Geological influence on hydrological diversity

Geological processes also play an important role in shaping the hydrological diversity. The relatively low sediment input (when compared with the westernmost Amazon), and paired subsidence and accommodation space in the central Amazon [112], caused relatively low slope and lithological diversity. Subsidence and basin infill have a strong influence on the megafan river landscape. Tectonic activity is another geological process shaping hydrological networks. For instance, river channels contain long straight segments, orthogonal junctions or orthogonal shifts in their course as a result of tectonic activity [91–93], or the channels are abandoned as a result of a tectonic mega-capture [93], enabling the creation of hydrologically diverse areas like the Anavilhanas Archipelago [88]. The presence of megafans might also contribute to the patch of high hydrological diversity located in southeast Peru, close to the border with Bolivia.

Hydrological diversity may also be derived from the chemical composition of water. Amazon rivers are generally categorized using the concept of whitewater, blackwater and clearwater rivers [145], a system of characterization and classification of rivers according to their chemical properties (figure 6*b*). The chemical composition is mainly controlled by the rock type present in a drainage basin that comes into contact with the water and may subsequently leach minerals into the water, and weathering rates [147–151]. It should be noted that the indigenous peoples of the Amazon have traditionally distinguished between river types using a more elaborate classification based on colour [152], which could aid the interpretation of the geological and geomorphological processes that shape the hydrology of the ADB.

Another hydrogeochemical research concerns the occurrence and source of toxic minerals in rivers, including but not limited to (heavy) metals such as mercury, arsenic, manganese, copper and iron [132,153–157]. Though human activity is often seen as a source of pollution, research has shown that human activity is only partly to blame for the presence of polluting minerals [132,155], or that all pollution seems to be geogenic (i.e. originating from the natural environment) [153,154,156,157]. Moreover, the chemical characteristics of water are strongly influenced by seasonal variations [154]. The hydrological regime in the ADB is not static; it is characterized by periodical flooding. This is described as the flood pulse concept [32], which varies across a climatological gradient [34]. Flood pulses lead to variable environmental conditions within a river–floodplain system, and may change the geomorphology, edaphic properties and redistribute nutrients [32]. Although the hydrogeochemical composition is not discussed in the context of hydrological diversity or geodiversity, variety in hydrogeochemical composition of rivers and fluctuating hydrological regimes create a highly dynamic environment.

### (d) Pedological diversity

**Figure 7.**
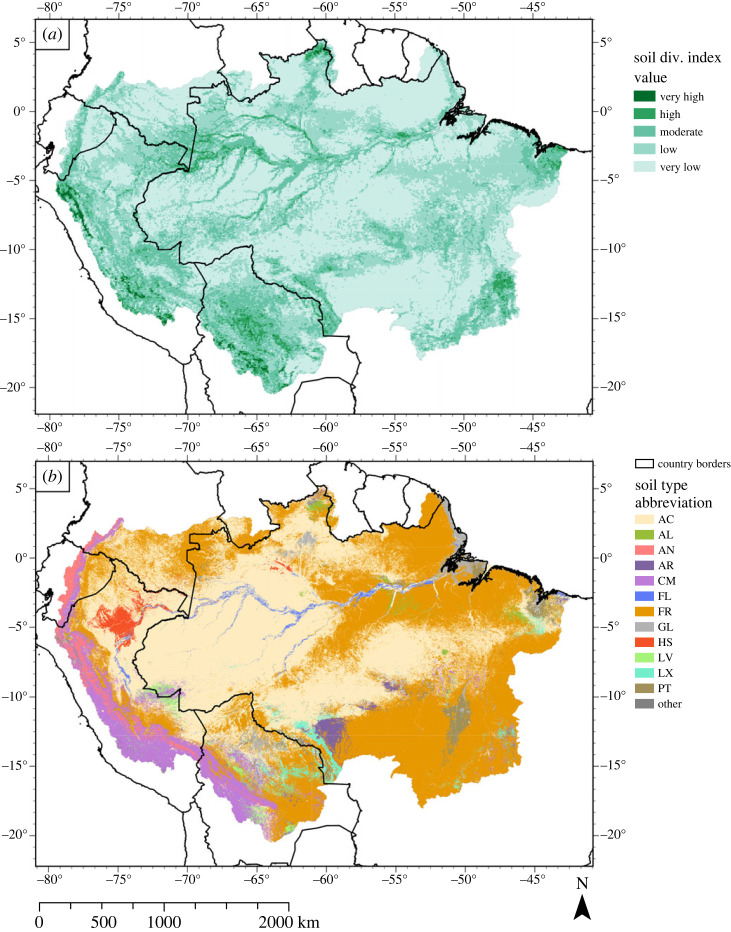
(*a*) Distribution of soil diversity in the ADB. Each 10 km × 10 km raster cell was awarded a score, based on the soil variety in that cell. Index classes (very low to very high) were created using a Jenks natural breaks classification (see Polman *et al*. [40] for detailed information). (*b*) Map showing the most likely soil type across the ADB. It serves as the source data for the soil diversity map. The data are retrieved from Hengl *et al*. [44]. AC, Acrisol; AL, Alisol; AN, Andosol; AR, Arenosol; CM, Cambisol; FR, Ferralsol; GL, Gleysol; HS, Histosol; LV, Luvisol; LX, Lixisol; PT, Plithisol; other, other soil types that were distributed too sparsely to be viewable. (Online version in colour.)

Soil formation depends on time, parent material, topography, climate and organisms [158]. This implies that soil diversity has tight relations to the other geodiversity components. The soil diversity index map (figure 7*a*), the lithological diversity index map and the slope diversity index map therefore do show some similarities. The soil diversity distribution is less sharply expressed than the lithological (figure 4*a*) and slope index (figure 5*a*). However, we observe a stretch of moderate to very high soil diversity located in the AOB and the adjacent megafans, where lithological and slope index classes are also moderate to very high. By contrast, the Amazon Craton is clearly defined in both the slope and lithological diversity index maps, but it is hardly distinguished in the soil diversity index map. Only a small belt of moderate to high soil diversity index classes is visible across the Central Brazilian Shield, and widening towards the Tocantins-Araguaia river system. The São Francisco Craton, however, is clearly defined by values of moderate to very high diversity in both the soil diversity map, as well as the lithological and slope diversity maps. The overlap between high diversity counts in geological, geomorphological and soil diversity is in agreement with Val *et al*. [34], who state that variations in parent material (i.e. lithological diversity) and geomorphology (i.e. slope diversity) are the main factors that influence local soil diversity.

Moderate to very high soil diversity index classes occur when lithological and slope diversity is low, such as at the Brazilian–Colombian–Peruvian border, and along the channels of the major rivers of the SA river system east of the Brazilian border. These are also areas with predominantly high to very high hydrological diversity classes, which suggest that hydrological diversity may contribute to higher soil diversity. The distribution of areas with high soil diversity index classes seems to correspond to the distribution of areas with high geodiversity index classes. Exceptions are the areas containing very high geodiversity index classes on the Guiana Shield, and the extent of moderate to very high geodiversity index classes on the Central Brazilian Shield (though these areas do contain overlap with moderate to high soil diversity index classes). Soil diversity patterns seem to generally resemble the diversity scores of the other geodiversity components.

#### (i) Differentiation of soil types

The mutual influence between geology, geomorphology, hydrology and soil type, together with time, have caused the diversification of soil types in the ADB. An example of this are the *terra firme* lower plateaux in the western ADB [159]. The uplift of the Andes, together with faulting activity changed the local drainage networks, and allowed for the differentiation of parent material, an important soil forming factor. Subsequent climatic changes then caused a further soil differentiation [159]. In addition, soil texture development, a result of parent material, weathering rates, geomorphological processes and time, has been suggested to be a further factor to differentiate the amounts of carbon content in the soil [160]. Ruokolainen *et al*. [161] assigned the unique geological history as explaining factors of the set of edaphic characteristics they identified in the Peruvian Amazon.

Time and topographic stability (related to geological stability), combined with high temperatures and wet conditions have caused deep soil profiles and intense leaching of nutrients from parent materials [162]. Typical soil types associated with these conditions are acrisols and ferralsols, which dominate the eastern and central Amazon (figure 7*b*). Acrisols are strongly weathered soils with a low base saturation, owing to a warm and wet climate [163]. Ferralsols are also deeply weathered soils that are typical for the continental shields of South America and generally poor in nutrients such as phosphorus and some 20 micronutrients [163].

Areas close to the AOB contain younger, less weathered soils of low pedogenetic levels (i.e. a low degree of soil development) such as cambisols, which are related to favourable soil fertility indicators such as a high nutrient content [162]. Other common soils of low pedogenetic levels are fluvisols, young soil found in fluvial, lacustrine and/or marine deposits [163], and gleysols which form under prolonged periods of groundwater saturation [163]. Gleysols and fluvisols are mainly found along the channels of rivers, in coastal areas, and in the Amazon river delta. Fluvisols and gleysols are especially present in floodplains and tidal areas. These soil types relate to the flood pulse concept [32], which suggests an added value of assessing soil diversity in geodiversity analyses.

An important part of soil research in the ADB is the mapping of geochemical background values of (potentially toxic) elements such as mercury, rare earth elements, iron, cadmium, nickel, lead, cobalt, chromium and zinc [164–168]. All articles mention geogenic sources as the primary factor for the concentration of chemical compounds in the soil. Input from anthropogenic sources such as mining is limited [169]. However, higher concentrations of macro- and micronutrients deeper in the soil suggest a closer connection to the underlying parent material [168]. Higher abundance of elements in soils atop metavolcanic, mafic and ultramafic rocks was found in the Carajás Mineral Province [165]. These findings seem to indicate that geological diversity is an important factor in governing soil properties.

## 5. Synthesis and outlook

The aim of this study was to present a comprehensive overview on how geodiversity patterns are formed across the ADB. We did this by analysing a global map of geodiversity metrics and by conducting a systematic literature review. And also, by assessing where patterns in maps agree with the literature and establishing which possible amendments to the global geodiversity maps would more accurately reflect geodiversity in the ADB.

### (a) Key characteristics of geodiversity in the Amazon drainage basin

**Figure 8.**
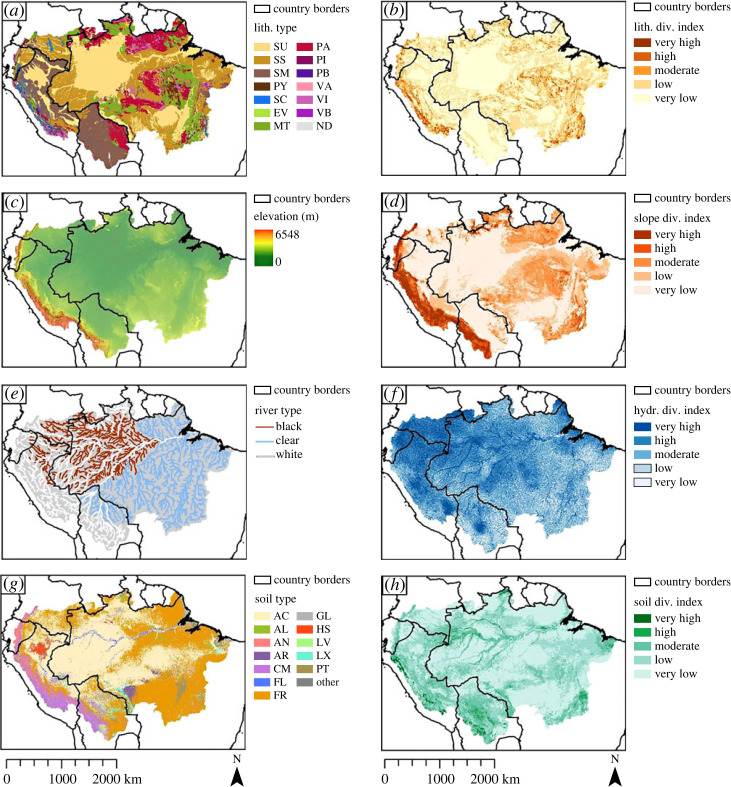
Overview of all geodiversity components and their base map. (*a*) Lithological diversity index, (*b*) most likely lithology, (*c*) DEM of the ADB, (*d*) slope diversity index, (*e*) river types of the major rivers in the ADB, (*f*) hydrological diversity index, (*g*) most likely soil type and (*h*) soil diversity index. For data sources and legend abbreviations, see original figures. (Online version in colour.)

After assessing the geodiversity map along with its components, it has become apparent that lithology and geological structures are the dominant factors controlling geodiversity in the ADB. Especially two regions stand out, namely the AOB and the western Amazon, and the cratons in the eastern Amazon. The patterns visible in the lithological diversity index map (figures 4*a* and 8*a*) are clearly recognizable in the geodiversity map (figure 2). Though the slope diversity index map exhibits similar patterns, they are linked to the orogenic and other tectonic processes that shaped the AOB and the craton-dominated region. The importance of geology in the ADB is underscored by the fact that most of the articles in the systematic literature review discussed geological geofeatures. Other literature cited the important role that geology played in the shaping of other abiotic components in the landscape. For instance, the geochemistry of water and soils was often attributed to the underlying lithology, and papers pertaining to geomorphology attributed certain landscape components to (neo)tectonic processes, that in turn influenced drainage patterns and soil formation processes.

A third region that stands out for its geodiversity in the ADB is the SA river system, which strongly contrasts with the two geodiversity units described in the previous paragraph. The river system cannot be explained by the patterns in lithology and slope diversity, as it has a low slope diversity and largely consists of one type of substrate (unconsolidated sediment). Instead, the high values of geodiversity can be attributed to hydrological diversity formed by alluvial fans, river channels and floodplains. Moderate to very high hydrological diversity index classes are mainly concentrated around the stream channels of the major rivers of the ADB, which have high hydrological index classes, in an area otherwise characterized by low geodiversity index classes. Though few articles assessed the hydrological diversity, numerous articles discussed important fluvial geomorphological processes that dominate the landscape. We therefore conclude that after geology, hydrology is the most important factor controlling geodiversity.

The distribution of soil diversity index classes largely coincides with the spatial distribution of the global geodiversity index classes. It is likely that the geodiversity index classes are amplified by the soil diversity index classes, as moderate to high soil diversity classes are mainly found in areas that already contain high lithological and slope or hydrological diversity index classes. As soil formation is dependent on parent material, topography and climate (which also included the availability of water) and organisms [158], this relationship is unsurprising. It must be noted that assessment of soil types in the ADB revealed additional information about important hydrological processes that did not appear in the hydrological diversity map. The occurrence of fluvisols and gleysols depends on the presence of water, but this presence may be variable (such as the occurrence of fluvisols in old floodplains or gleysols in areas that experience variable groundwater levels). These hydrological geofeatures are closely related to the flood pulse system that characterizes the ADB [32], but are not visible in the hydrological diversity index map.

### (b) Alternative approaches to geodiversity analysis in the Amazon drainage basin

Maps and literature both agree on the importance of geology and hydrology for geodiversity in the ADB, but several potential adjustments in the method of assessing geodiversity have been identified. Some geodiversity assessments included fossil sites and mineral ore and deposits as important geofeatures [35,38]. Especially the mapping of mineral deposits might give additional information, as articles have repeatedly reported that the (hydro)geochemistry of rivers and soils is affected by the presence of ore deposits. Moreover, it is important to note that although mining activities can increase the degradation risk of geosites [53], they also create artificial stratotypes and new surface topographies [170] that locally might increase geodiversity. It would be interesting to research this relationship further, especially in mineral rich sites as the Carajás Mineral Province, which is also home to many special geological features as reported in §4a.

Other approaches to geodiversity mapping have been identified in geomorphological papers. As fine scale geomorphological features are not available on a large extent for the ADB, the slope diversity index was used as a proxy for geomorphology. However, when assessing the slope diversity index and DEM maps carefully (figures 5*a*,*b* and 8*a*,*b*), it is clear that slope diversity alone fails to distinguish between different geomorphological units, as was the case with the Xingu river headwaters. Geomorphon analysis is a promising method that could solve this problem, as it is a fully automated quantitative analysis that generates different archetypes of particular terrain morphology [137]. It has been applied successfully to the Tocantins-Araguaia catchment in the ADB [136], and to the entire country of Poland [137]. In addition, there is an increase in the availability of high-resolution DEMs, e.g. from high-resolution LiDAR data, which may form a basis to extract fine-scale geomorphological maps, either using the geomorphons approach or using a geographic object-based image analysis (GEOBIA) approach [171,172]. Though computational efficiency can be a problem when using GEOBIA for large datasets, segmentation algorithms are improving with the aim of higher degrees of accuracy, automation and computational efficiency [172].

As already mentioned, mapping the hydrogeochemistry of rivers could provide an interesting new outlook for assessing geodiversity in the ADB, and is not only limited to the mapping of potentially toxic elements. Mapping river types, as is shown in figure 6*b* (i.e. whitewater, blackwater and clearwater rivers) [22], or by other hydrological, physico-climatic and geomorphological characteristics [45] could also provide new insights. Another possible angle from which to approach hydrological diversity is to also address fluctuations in water distribution. Currently, such hydrological geofeatures can only be inferred from the distribution of soil types that are strongly influenced by the presence of water, as is discussed in the previous section. Variability in water distribution could be incorporated into grid-based analyses, for example, by distinguishing areas that are never flooded, hardly ever flooded or frequently flooded. Including geochemical and hydrological regimes in geodiversity analyses is especially interesting for geodiversity research related to biodiversity, as water and nutrient availability is crucial in determining which species can successfully establish in an area.

All these alternative approaches call for a standardization of geodiversity classification across areas and scales. A first attempt is presented by Hjort *et al*. [173], who developed a first taxonomy of geodiversity. This taxonomy is a simple, adaptable, transferable system for classifying geodiversity in six hierarchical levels. Such a system could potentially be applied in mapping and quantifying geodiversity in the ADB, and help to explore genetic relationships and finding and explaining physical mechanisms, e.g. between slope, lithology, soils and hydrology.

### (c) Methodological challenges

In our assessment, we evaluated the main characteristics of geodiversity in the Amazon. However, there are several methodological issues that need to be mentioned. Firstly, the diversity index maps were analysed manually, so only large overlapping patterns and relationships between geodiversity components were assessed. This issue can be resolved by performing a quantitative analysis based on cell statistics, which generates statistical information regarding the contribution of each geodiversity component to the geodiversity index and could provide new insights into how geodiversity components interact to create areas with high geodiversity.

Secondly, there is the issue of scale. Geodiversity assessments are prone to scaling issues [174]. The geodiversity index maps that are at the basis of this review are extracted from a global scale dataset [41]. By using finer scale maps, one could obtain different geodiversity index distributions. One example is the discrepancy between the geodiversity ranking of the Xingu river basin between the maps made on a local, catchment scale [38] and a global scale [41]. Another is the seemingly flat areas in Solimões and Amazonas sedimentary basins, while in truth, one can still successfully map different topographical units based on elevation [125]. The difference lies in the scaling: the entire ADB varies between 0 and more than 6500 m in elevation, whereas the range of elevation in the basins is considerably smaller.

A different issue pertaining to scale is the fact that there are no harmonized, fine-scaled, global lithology, geomorphology, hydrology or soil datasets. The soil basemap is based on predictions calculated from relatively sparse sampling points [44]. This carries the risk of being inaccurate, especially as one assesses areas on a finer scale. De Paula Silva *et al*. [38] show that the geodiversity distribution can change significantly if more accurate, finer scale lithological data are used. This presents an opportunity for further refinement of the results of this research: the creation of a geodiversity index specific to the ADB, by harmonizing local lithology and soil maps and including some of the suggested geodiversity components listed above.

Thirdly, caution is advised when only using maps to select possible geosites in the ADB. Based on the geodiversity index map, it is most likely that suitable geosites can be found in the AOB, the cratons, and along the areas with high hydrological diversity in the sub-Andean foreland basins, the Solimões and Amazonas sedimentary basins. However, it is important to note that a previous study noticed that areas that ranked low in geodiversity were still of high ecological interest due to important lithological and geomorphological features found there [35]. Thus, simply using a grid-based approach is insufficient, and it is advised to combine a grid-based method using the approach as suggested by Brilha [19], which can be regarded as a qualitative–quantitative method as proposed by Zwolinski *et al*. [175].

### (d) Geodiversity as driver of biodiversity

A large body of literature highlights the influence of one or more geodiversity components on biodiversity patterns in the ADB. In particular, geology was seen as an important factor controlling floristic diversity patterns, as geology controlled edaphic properties [29,176–185]. For instance, the net primary productivity, the net amount of carbon dioxide captured by plants and converted into organic compounds [186] in the ADB, is influenced by phosphorus availability [187], which in turn depends on the parent material and to what degree the soil has weathered [162]. The floristic gradient, as a result of edaphic gradients, has also been shown to influence avian species composition [30]. Geology also affects species diversity due to tectonic processes, as tectonic subsidence and the formations of faults cause topographic gradients influencing vegetation diversity and distribution patterns [138,139]. Topography and hydrology also affect species richness and composition [188], and river type has also been shown to influence species richness, as forests along blackwater rivers (called igapó) generally contain a higher number of species and a different species composition compared to whitewater forests (called várzea) [189]. Naturally, fish species distributions are also dependent on the water geochemical properties [28,190].

Geodiversity and biodiversity both thus seem to be controlled by geology and hydrology, which could serve as a starting point into the assessment of the link between biodiversity and geodiversity in the ADB. However, the research mentioned above does not imply that the link between geodiversity and biodiversity is clear-cut. For instance, research has shown that fish diversity and tree diversity are highest in areas that are considered to be low in geodiversity in this study (i.e. the central Amazon, away from the SA river system, characterized by low to very low geodiversity index classes) [191–193]. This suggests that biodiversity in the ADB is not always related to geodiversity. Moreover, biotic interactions and habitat structures should not be overlooked when explaining biodiversity patterns of related species based on geodiversity patterns; competition, food resources or other inter-specific interactions could also contribute to determining the species composition gradient across a landscape [190]. In addition, in the Andes, elevation, temperature and precipitation should also be considered as abiotic driving factors behind biodiversity patterns [31,194], which are factors that have not been considered in this review at all.

## 6. Concluding remarks

As yet, geodiversity is an understudied subject in the ADB, but a recent global geodiversity map provided a useful base for identifying the main factors that influence geodiversity. The global geodiversity index map revealed that the geodiversity index classes were especially high in the AOB, the Amazon, São Luís and São Francisco cratons, and around dense drainage systems and large river channels. Close examination of each of the geodiversity components showed that the ADB is strongly influenced by geology and hydrology, which was a recurring explaining factor in the distribution of geomorphological features and soil types in the literature. Moreover, geological diversity is defined not only by lithological diversity, but also by geological structures that are the product of former, and still active, tectonic processes. In our review, we also identified several additional approaches to assessing geodiversity, such as the mapping of geomorphons or using GEOBIA in order to assess geomorphological diversity, and the mapping of geochemical properties in water and edaphic properties of soils. These suggestions could not only improve the accuracy of geodiversity mapping in the ADB, but also improve the understanding of geodiversity and biodiversity relationships, which are characterized by geological diversity and hydrogeochemical and edaphic gradients.

Further investigation of the precise relationship between geology, geomorphology, hydrology and soil is needed to gain a deeper understanding of the physical mechanisms that contribute to geodiversity in the ADB. Quantitative methods such as cell statistics and using finer scale data by harmonizing local lithology and soil maps, in combinations with the previously suggested amendments to the mapping of geomorphology and hydrology, can aid in achieving this goal. Using these methods, a geodiversity index map specific to the ADB can be created, which enables the localization of potential geosites. The conservation of geosites that are characteristic of the processes that influence geodiversity will warrant the protection of important scientific and educational information for generations to come.

## Data Availability

Additional data can be downloaded as an ArcGIS Pro Project File, consisting of all the maps shown in this review (AmazonGD.ppkx). Supplementary material is available online [51].
